# A Highly Integrated BOTDA/XFG Sensor on a Single Fiber for Simultaneous Multi-Parameter Monitoring of Slopes

**DOI:** 10.3390/s19092132

**Published:** 2019-05-08

**Authors:** Feng Li, Weigang Zhao, Hongbin Xu, Shupeng Wang, Yanliang Du

**Affiliations:** 1 School of Civil Engineering, Wuhan University, Wuhan 430072, China; shupeng2hao@163.com; 2 Structural Health Monitoring and Control Institute, Shijiazhuang Tiedao University, Shijiazhuang 050043, China; zhaoweig2002@163.com (W.Z.); xuhongbin@semi.ac.cn (H.X.); du_yanliang@163.com (Y.D.); 3 Key Laboratory of Structural Health Monitoring and Control, Hebei Province, Shijiazhuang 050043, China

**Keywords:** slope, landslide monitoring, fiber optic sensor, multiparameter monitoring, hybrid sensing technology

## Abstract

A highly integrated sensing technology, combining a stimulated Brillouin scattering-based distributed sensor with XFG (fiber Bragg grating (FBG) and long-period fiber grating (LPFG)) sensors on a single fiber, is proposed for the simultaneous measurement of fully distributed and multiple discrete dynamic strains/temperatures. A multiparameter monitoring scheme for slope safety is developed using this integrated sensing technology. An indoor simulation test is carried out to verify its ability to simultaneously monitor a slope’s surface displacement, an anchor reinforcement’s axial force, and rockfall vibration. The experimental results show that distributed static strain and discrete dynamic strain can be well-measured simultaneously with little interference. The results also demonstrate the XFG sensors’ capability for multi-type and multipoint multiplexing. In addition, the proposed hybrid sensor system has potential for the monitoring of multiple slope parameters simultaneously.

## 1. Introduction

Landslides are one of the three major geological disasters that threaten human life and property throughout the world. In particular, transport infrastructures that have expanded into mountain areas in recent decades are often accompanied by artificial slopes. These artificial slopes have become one of the most significant threats to transport infrastructures.

Landslide monitoring and reinforcement play an important role in preventing or mitigating hazards. Various methods have been introduced into landslide monitoring and detecting, including Global Positioning System (GPS) [1,2], remote sensing [3], interferometric synthetic aperture radar (InSAR) [4,5], time domain reflectometry (TDR) [6], conventional inclinometer [7,8] and ground-penetrating radar (GPR) [9,10]. However, these methods mainly focus on the detection of a single parameter, namely surface monitoring or subsurface monitoring, making it difficult and costly to develop an integrated warning system for hazard management. At the same time, fiber optical sensing technologies have also been widely used in landslide monitoring due to their small size, high resolution, and in-site monitoring and multiplexing capabilities [11,12,13,14]. Previous studies have established that surface deformation [15,16], subsurface deformation [17,18,19,20], reinforcements [21,22,23,24,25], the strain field [26,27,28,29], soil pressure [30,31], rockfall [32], and hydraulic characteristics [33,34,35] can be well-measured using fiber Bragg grating (FBG) sensors and Brillouin-based distributed sensors. Because of the complicated mechanisms and diverse triggering factors, information on multiple parameters is crucial in a practical application to evaluate the safety of a slope accurately [6,36]. According to the literature, it is possible to establish an integrated monitoring system based on optical fiber technology [37,38,39]. As we know, FBG sensors feature quasi-distributed, accurate, real-time, and dynamic measurements. Brillouin-based distributed sensors have advantages over FBG sensors in long-distance and large-scale measurement, but most widely apply in static measurements. Therefore, a combination of an FBG sensor and a Brillouin-based distributed sensor is of great significance to provide an early warning system for landslides. Shi et al [37] utilized both an FBG sensor and a BOTDA (Brillouin optical time distributed analysis) sensor to monitor slopes. However, two separate optical fibers were adopted in their solution. Other attempts have been made to combine the two technologies on a single fiber. Ou et al [40,41] proposed a system that integrated FBG and BOTDA/R sensors on a single fiber. They used an optical switch or coupler to realize the combination. A similar scheme was also applied to transmission line [42] and corrosion [43] monitoring. However, separate interrogators for the BOTDR/A and FBG sensors were used in these applications. On the one hand, separate interrogators make the monitoring system complicated and costly. On the other hand, BOTDR/A and FBG interrogators cannot work synchronously when using an optical switch. Although synchronous measurements can be performed using a coupler for BOTDR and FBG interrogators with little interference, BOTDA and FBG interrogators cannot work synchronously because a BOTDA interrogator features double-ended loop testing and an FBG interrogator features a wavelength-swept laser source without a filtering function at the end. One of the two laser beams is led into a photo-detector of the FBG interrogator directly, leading to saturation and a reduction in the signal-to-noise ratio (SNR) of the FBG demodulation system. Furthermore, the peak reflected from the FBG sensor would not be found or detected accurately. At the same time, the reflected signal from the FBG sensor may also be led into the BOTDA sensor’s detector, causing interference with the BOTDA sensor’s measurement. So, it is of great significance to develop an integrated system in which the two sensors can work simultaneously without interference.

In this study, we propose a highly integrated BOTDA/XFG sensor on a single fiber. This hybrid system realizes synchronous BOTDA and XFG sensor measurements using a shared optical source, a receiver unit, and a single fiber. First, we investigated the hybrid system’s performance and discussed its capability for multi-type and multipoint multiplexing. Then, we developed a scheme for the monitoring of multiple slope parameters using this hybrid system and experimentally verified its feasibility in simultaneously monitoring a slope’s surface displacement, a reinforcement’s axial force, and rockfall vibration. Moreover, we discuss some future work and key problems that need to be solved to improve this integrated system for slope monitoring.

## 2. The Hybrid BOTDA/XFG Sensing Technology

### 2.1. XFG Sensor

The XFG sensor includes a fiber Bragg grating sensor and a long-period fiber grating (LPFG) sensor, which both feature a spatially periodic modulation of the refractive index along a fiber’s core. When light from a broadband source is coupled into an FBG sensor, a narrow spectrum with a special wavelength is reflected in the core mode. This special wavelength corresponds to the central wavelength of the FBG sensor. However, when broadband light is transmitted into an LPFG sensor, the wavelength that meets the LPFG sensor’s resonance condition is coupled into the cladding of the fiber, forming the cladding code. In general, a reflection spectrum and a transmission spectrum are obtained for the demodulation of an FBG sensor and an LPFG sensor, respectively.

When an FBG or LPFG sensor is subjected to external mechanical or thermal variation, the reflected or transmitted peak wavelength will shift. The influence of strain and ambient temperature on the grating period and the refractive index can be expressed as follows [44]:

(1) $$\Delta \lambda_{B}=\lambda_{B}\left[\left(1-P_{e}\right)\Delta \varepsilon +\left(\alpha +\xi \right)\Delta T\right]$$

where Δ*ε* is the strain variation, Δ*T* is the temperature change, *α* is the coefficient of the thermal expansion, *ξ* is the thermo-optic coefficient, and *P*_e_ is the strain-optic coefficient.

As we know, an LPFG sensor is more sensitive to changes in the refractive index than a normal FBG sensor, so it is more suitable for humidity and moisture detection. An LPFG-based sensing technology has been developed for humidity and moisture monitoring [45].

### 2.2. Distributed Optical Fiber Sensor

In a BOTDA sensor, a continuous wave (CW) probe and a modulated pump wave counter-propagate along the sensing fiber in order to induce stimulated Brillouin scattering light (SBS). A maximum power transfer from the pump wave to the probe wave would occur when the frequency difference between the pump and probe waves matches the frequency associated with the vibration of a crystal lattice. The corresponding frequency difference is referred to as the Brillouin frequency. The Brillouin frequency *ν*_B_ depends on the properties of the optical fiber, which typically ranges from 9 GHz to 13 GHz for a silica-based single mode optical fiber and can be given as follows:

(2) $$\nu_{B}=\frac{2\nu_{0}}{c}nV_{a}$$

where *ν*_0_ denotes the frequency of incipient light, *n* denotes the refractive index of the optical fiber, *V_a_* denotes the speed of the acoustic wave in the fiber, and *c* denotes the speed of light in a vacuum. The speed of the acoustic wave is calculated by [44]:

(3) $$V_{a}=\sqrt{\frac{(1-\mu )E}{(1+\mu )(1-2\mu )\rho }}$$

where *μ*, *E*, and *ρ* denote the optical fiber’s Poisson’s ratio, Young’s modulus, and density, respectively.

Therefore, the Brillouin frequency changes with the strain and temperature applied to the optical fiber. For a relatively small change in strain and temperature, the Brillouin frequency shift Δ*ν*_B_ can be given as follows [46]:

(4) $$\Delta \nu_{B}=C_{\varepsilon }\Delta \varepsilon +C_{T}\Delta T$$

where *C*_*ε*_ and *C*_*T*_ denote the strain and temperature sensitivity coefficients, respectively.

The corresponding position of strain or temperature changes in an optical fiber can be measured using the optical time domain reflectometry (OTDR) technique.

### 2.3. The Hybrid BOTDA/XFG Sensing Technology

**Figure 1:**
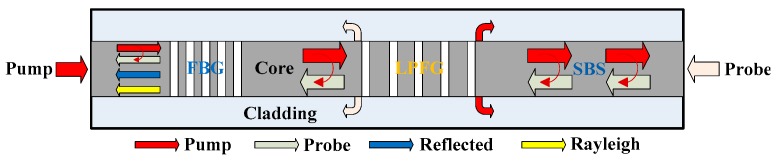
A schematic diagram of the hybrid BOTDA/XFG sensor on a single fiber.

The basic principle of the hybrid BOTDA/XFG sensor is shown in Figure 1. A pulsed pump light and a continuous wave probe light can be used for a BOTDA-based sensor. At the same time, these counter-propagating lights may also be used as the light source for a reflection-based FBG sensor and a transmission-based LPFG sensor, respectively. In order to realize hybrid sensing, two key problems should be solved. One is the intensity adjustment that is required to pump light into both the BOTDA sensor and the XFG sensor simultaneously. The other is multiplexing and demultiplexing to avoid interference between the stimulated Brillouin signal and the XFG signal.

Due to the double-ended loop testing feature of a BOTDA sensor, the signal detected at the pump end is the superposition of probe light and reflected light. If the propagation loss is not considered, the signal intensity of the XFG sensor detected at the pump end, which is defined as P_dect_, can be expressed by the following formula:

(5) $$\left\{ \begin{array}{l} \begin{array}{c} {P^{\prime }}_{\mathrm{dect}}=P_{\mathrm{probe}}(1-r^{\prime })+P_{\mathrm{pump}}r^{\prime }\begin{array}{c} \;(\begin{array}{cc} \mathrm{for} & \begin{array}{c} \mathrm{FBG}\begin{array}{cc} & \mathrm{sensor} \end{array} \end{array}) \end{array} \end{array} \end{array}\\ \begin{array}{c} {P^{\prime \prime }}_{\mathrm{dect}}=P_{\mathrm{probe}}(1-r^{\prime \prime })\begin{array}{c} \;(\begin{array}{cc} \mathrm{for} & \begin{array}{c} \mathrm{LPFG} \end{array}\begin{array}{cc} & \mathrm{sensor} \end{array}) \end{array} \end{array} \end{array} \end{array}\right.$$

where P′_dect_ and P″_dect_ denote the signal intensity from the FBG sensor and the LPFG sensor, respectively, P_probe_ and P_pump_ are the probe light intensity and the pump light intensity at the XFG sensor’s location, respectively, and r′ and r″ are the reflectivity of the FBG sensor and the LPFG sensor, respectively.

Considering the background signal of the probe light detected by the XFG interrogator, the difference between P_dect_ (at the working wavelength of the XFG sensor) and P_probe_ (near the working wavelength of the XFG sensor) is needed. At the same time, the greater the difference, the better the SNR of the XFG interrogator. In this way,

(6) $$P=10\mathrm{lg}\frac{P_{dect}}{P_{probe}}$$

where *P* is the power difference between the probe light’s intensity and the XFG sensor’s signal intensity, which is defined in dB.

As for an ordinary LPFG sensor, high reflectivity (more than 90%) usually makes P lower than −10 dB. For an FBG sensor with high reflectivity, only when P_pump_/P_probe_ is large enough can a better SNR be obtained. In contrast, the value of P will be close to zero when P_pump_ approaches P_probe_. Therefore, if a power difference between the pulsed pump light and the CW probe light at various wavelengths exists, the CW probe signal containing BOTDA sensing information and LPFG sensing information and the reflected signal containing FBG sensing information and Rayleigh sensing information can be detected simultaneously at the pump end.

As we know, in a BOTDA system, the SBS signal contains a Stokes component and an anti-Stokes component. Both components will shift with strain and temperature. According to the principle in Section 2.2, the frequency of the Stokes component ν_s_ and the anti-Stokes component ν_as_ can be given by the following formula:

(7) $$\{\begin{array}{c} \begin{array}{l} \begin{array}{c} \upsilon_{s}=\upsilon_{0}-\upsilon_{B}-\Delta \upsilon_{B} \end{array}\\ \begin{array}{c} \upsilon_{as}=\upsilon_{0}+\upsilon_{B}+\Delta \upsilon_{B} \end{array} \end{array} \end{array}$$

where *ν*_0_ denotes the frequency of incident light, *ν*_B_ denotes the Brillouin frequency of the optical fiber, and Δ*ν*_B_ denotes the Brillouin frequency shift (BFS) of the optical fiber with a change in strain and temperature.

Then, the corresponding wavelengths *λ*_s_ and *λ*_as_ of the SBS signal can be calibrated as follows:

(8) $$\left\{ \begin{array}{l} \begin{array}{c} \lambda_{s}=\frac{c}{n\upsilon_{s}}=\frac{c}{n(\upsilon_{0}-\upsilon_{B}-\Delta \upsilon_{B})} \end{array}\\ \begin{array}{c} \lambda_{as}=\frac{c}{n\upsilon_{as}}=\frac{c}{n(\upsilon_{0}+\upsilon_{B}+\Delta \upsilon_{B})} \end{array} \end{array}\right.$$

where *c* denotes the speed of light in a vacuum, and *n* denotes the refractive index of the optical fiber.

**Figure 2:**
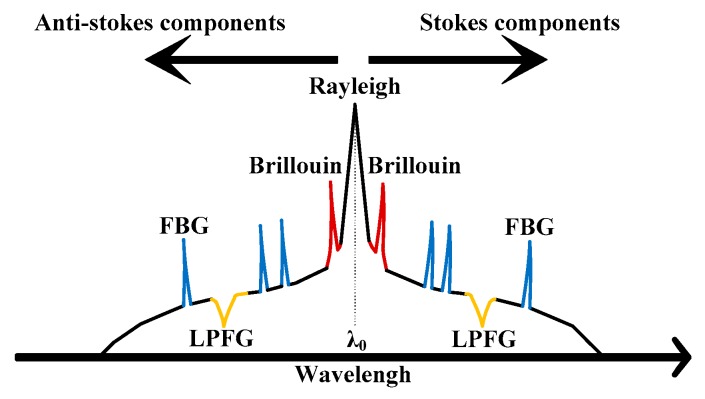
The spectral distribution of the hybrid BOTDA/XFG sensing technique.

In order to avoid interference between the SBS signal and the XFG signal, the working wavelength of the XFG sensor should be located beyond (*λ*_as_, *λ*_s_). Taking the basic parameters of the optical fiber and the ambient strain/temperature changes into consideration, the maximum wavelength shift of the SBS signal from incident light (*λ*_0_) is usually 100 picometers. When measuring stain or humidity, the reflected light from the FBG sensor and the transmitted light from the LPFG sensor can spread over a wavelength range of tens of nanometers, with the central wavelength near *λ*_0_. Once there is no overlap between the wavelength of the SBS light signal and the working wavelength of the FBG reflected light signal and the LPFG transmitted light signal, both the distributed strain/temperature and the pointed dynamic strain or humidity can be measured by using the wavelength-division multiplexing technology. The spectral distribution of the hybrid BOTDA/XFG sensing technique is schematically shown in Figure 2.

## 3. Experimental Setup

**Figure 3:**
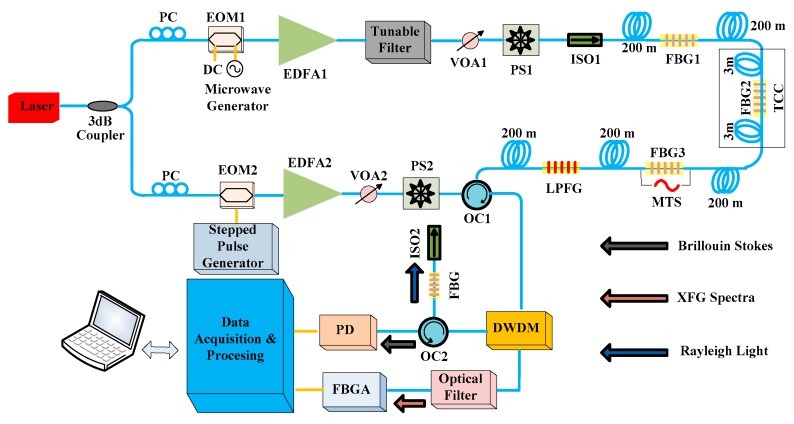
A schematic of the hybrid BOTDA/XFG sensing system.

Figure 3 shows the schematic of the hybrid BOTDA/XFG sensor system. The proposed scheme has the advantage of using a single narrowband DFB laser, which is exploited to simultaneously generate both the XFG pump for FBG sensor and LPFG sensor measurements as well as the Brillouin pump and CW probe for BOTDA sensor measurements.

In this scheme, a DFB fiber laser centered at 1548.52 nm with a narrow linewidth is used as the sensing source of the hybrid system. The output of this laser is divided into two branches using a 3 dB optical coupler. As shown in Figure 3, the lower branch is used to generate a pulsed pump light for the BOTDA sensor’s measurement, as well as to interrogate the FBGs. A Mach–Zehnder electro-optic modulator (EOM2), driven by a stepped pulse generator [47], is utilized to modulate the continuous wave to obtain pulsed light. The duration and amplitude of the pump pulse are 1 ns and 29 dBm, respectively. An Erbium-doped fiber amplifier (EDFA2) is used to amplify the pulse peak power at the fiber’s input. At the same time, an Erbium gain is generated in a broad wavelength range (used for interrogating the FBGs). A variable optical attenuator (VOA2) is used to adjust the optical power that is transmitted into the fiber. After that, the pulsed light is simply polarization-scrambled through a PS2 so as to avoid Brillouin polarization dependencies. It is then injected into the fiber under test through a three-port optical circulator (OC1).

The fiber under test is composed of ~1 km of SMF and an XFG sensor array that includes three FBG sensors (namely FBG1, FBG2, and FBG3) and one LPFG sensor. The 3 dB bandwidth and reflectivity of the FBG sensors are approximately 0.25 nm and 92%, respectively. The 3 dB bandwidth and reflectivity of the LPFG sensor are 2.75 nm and 95%, respectively. The XFG sensors are located at different positions along the fiber with different center wavelengths that range from 1530 to 1570 nm. Among them, FBG3, which is located at ~600 m, is driven by an MTS universal testing machine for pointed dynamic strain measurement. Meanwhile, a 6-meter-long fiber separated by FBG2 is utilized for distributed temperature measurement. This sensing fiber, located at ~400 m, is placed into a temperature-controlled cabinet (TCC).

In the upper branch, a CW probe light is generated as the detected signal for the BOTDA subsystem, as well as to interrogate the LPFG sensor. The probe light counter-propagates with the pump light in the fiber and finally travels back to the detector. A Mach–Zehnder electro-optic modulator (EOM1) is driven by a microwave (RF) generator and biased at the minimum transmission in order to generate optical double-sideband suppressed-carrier (ODSB-SC) modulation. Then, an Erbium-doped fiber amplifier (EDFA1) is employed to amplify the suppressed carrier and the double-sideband. After that, a tunable filter is used to remove the high-frequency sideband. A variable optical attenuator (VOA1) is used to adjust the optical power that is transmitted into the fiber, while a polarization scrambler (PS1) is employed to reduce polarization-induced gain oscillations. Finally, this probe light is coupled into the fiber under test after isolation.

At the receiver side, the probe signal (containing Brillouin and LPFG information) and the reflected signal (containing FBG and Rayleigh information) are then imported into the detection system through the optical circulator (OC1). A DWDM centered at 1548.5 nm with a bandwidth of ~1 nm is used after the optical circulator (OC1) imports the signals to separate the XFG spectra from the Brillouin Stokes component and the Rayleigh backscattered signal. The Brillouin Stokes component is then extracted using a narrowband FBG filter (centered at the pump wavelength with 6 GHz bandwidth) and an optical circulator (OC2). The signal reflected from DWDM is then imported into an optical band-pass filter with a center wavelength of 1548.5 nm and a bandwidth of 3.0 nm to further reduce the intensity of the Brillouin and Rayleigh components. Finally, the XFG spectra and the Brillouin Stokes component are respectively coupled into a fiber Bragg grating analyzer (FBGA, BaySpec, Inc.) and a photodetector. All these are controlled by a computer that is also used to acquire and process the measured data. This scheme combines the advantages of both PPP-BOTDA [48] with a spatial resolution of 10 cm in 1 km of sensing fiber and inline wavelength-division-multiplexed (WDM) XFG interrogation.

## 4. Experimental Results and Discussion

### 4.1. Spectral Characteristics and Capacity Of the Hybrid System

**Figure 4:**
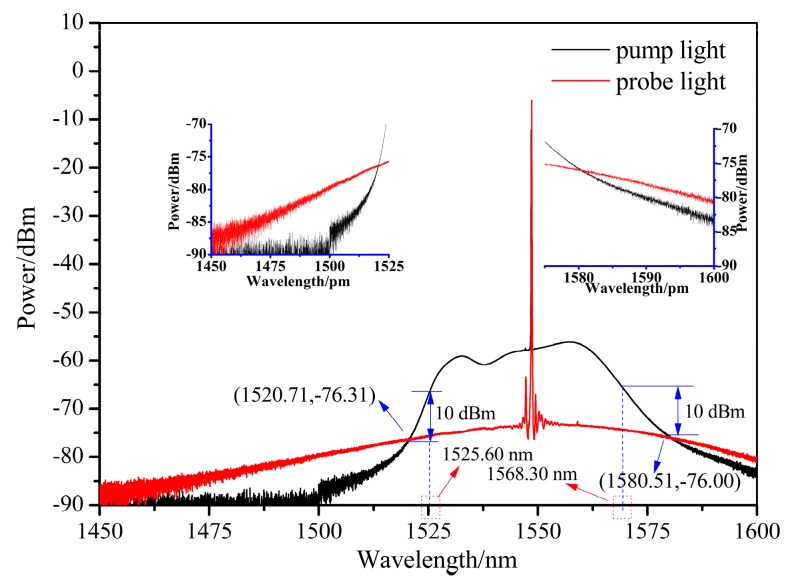
The emission spectrum of the hybrid system.

**Figure 5:**
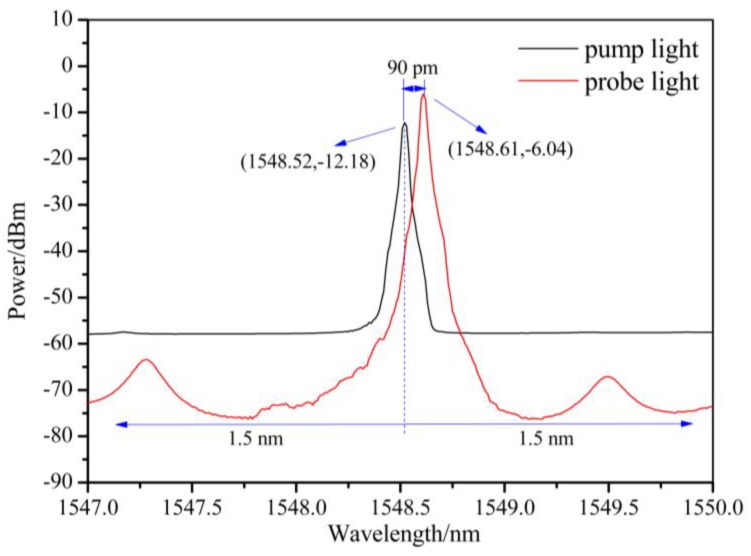
The frequency drift between the pump light and the probe light.

The emission spectrum of the hybrid system was measured by an optical spectrum analyzer (OSA) (ANDO AQ6317C) from 1450 to 1600 nm, as shown in Figure 4. From Figure 5, it can be seen that the probe light’s wavelength is 1548.610 nm, while pump light’s wavelength is 1548.520 nm. The spectral separation between the pump and probe light in this experiment is 90 pm (~10.84 GHz) when pumping at 1548 nm, which is approximately in agreement with the result observed in the Brillouin frequency spectrum.

**Figure 6:**
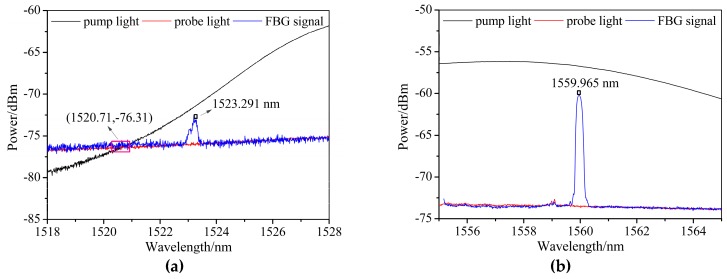
The signal-to-noise ratio (SNR) of XFG sensors with different center wavelengths: (**a**) from 1518 nm to 1528 nm; (**b**) from 1555 nm to 1565 nm.

The pump light and probe light have equal power at the wavelengths of 1520.71 and 1580.51 nm. When the wavelength is located at 1450–1520.71 nm and 1580.51–1600 nm, the power of the probe light is higher than that of the pump light. In contrast, the pump light has higher power than the probe light between 1520.71 and 1580.51 nm. As we know, the XFG sensors in an optical system feature a wavelength filtering function, so avoiding overlap with the working wavelength for the BOTDA sensor’s measurement is needed. As shown in Figure 5, 1.5 nm of red-shift and blue-shift are suggested from 1548.52 nm (the pump light wavelength) and 1548.61 nm (the probe light wavelength), respectively, to minimize the interference of BGS. At the same time, considering the superposition of the reflected and transmitted signals of the XFG sensor and the direct probe signal in the measured spectrum, the wavelength with a power difference (nearly 10 dB, see Figure 4) between the pump light and the probe light is needed. This has been verified in Figure 6, which demonstrates that an obvious difference exists in the SNR of the XFG sensor that is dependent on the power difference between pump light and the probe light. In summary, the wavelength for XFG interrogation is suggested to be located at 1525.60–1547.02 nm and 1550.11–1568.30 nm to obtain a better SNR.

### 4.2. BOTDA-Based Distributed Measurement

**Figure 7:**
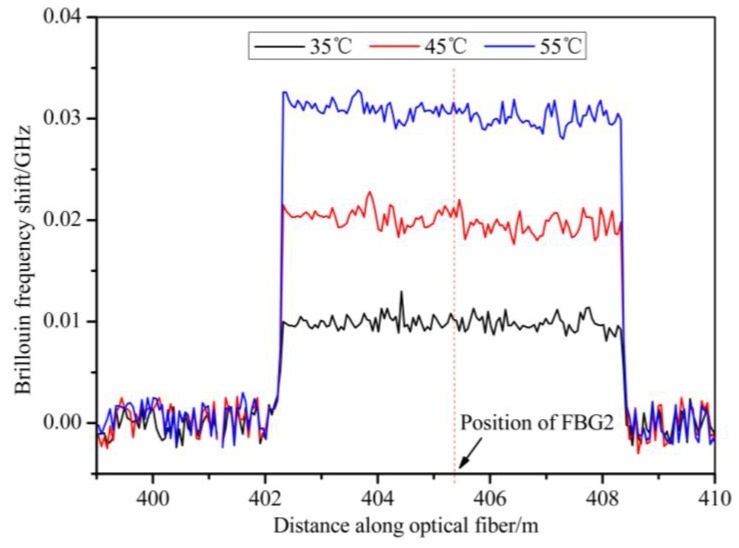
The Brillouin frequency shift (BFS) of the optical fiber with different temperatures.

The distributed temperature was measured along the fiber, of which 6 meters was placed in a TCC and the rest kept at room temperature. Then, the influence of an FBG sensor on BOTDA-based distributed measurement was studied. Figure 7 shows the Brillouin frequency shift of the fiber with different temperatures. In this experiment, the temperature of the TCC was set to be 35 °C, 45 °C, and 55 °C from room temperature. As shown in Figure 7, the distributed temperature measurement is almost not influenced by the FBG sensor. This was attributed mainly to the center wavelength of the FBG sensor being far away from the working wavelengths of both the incident light and SBS signal and the DWDM used as a Brillouin filter.

According to the standard deviation of the BFS (1.47 MHz) in Figure 7, a temperature resolution of 1.52 °C can be achieved with a temperature–BFS coefficient of 0.97 MHz/°C.

### 4.3. FBG-Based Pointed Measurement

**Figure 8:**
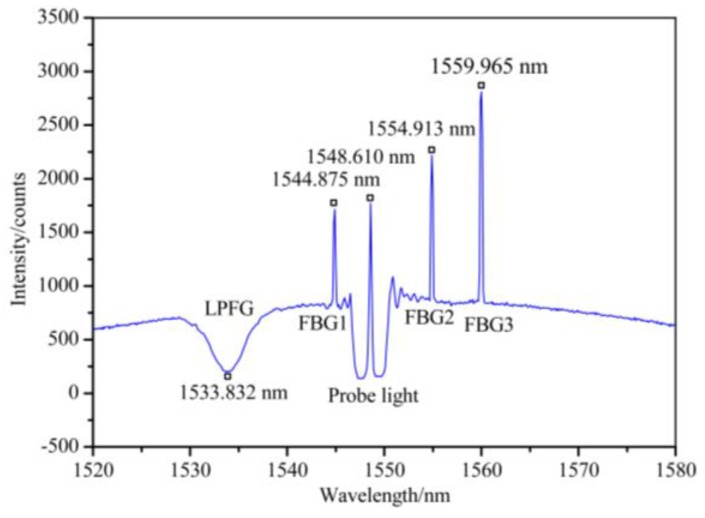
The measured spectrum of the XFG sensors.

Figure 8 shows the measured spectrum of the XFG sensors, which include one LPFG sensor and three FBG sensors, with different center wavelengths. The LPFG sensor is centered at 1533.832 nm, while the three FBG sensors are respectively centered at 1544.875 nm, 1554.913 nm, and 1559.965 nm. Due to the DWDM, the SBS and Rayleigh signals are separated to avoid spectral crosstalk. The XFG signals are superimposed by the probe’s incident light, which has been significantly attenuated using the DWDM and the subsequent band-pass filter to avoid detector saturation in the FBGA. This validates the multiplexing of the XFG sensors in a single fiber. It also proves that, in the hybrid system, the Bragg reflected signal, the LPFG transmitted signal, and the Brillouin scattering signal do not interfere with one another.

**Figure 9:**
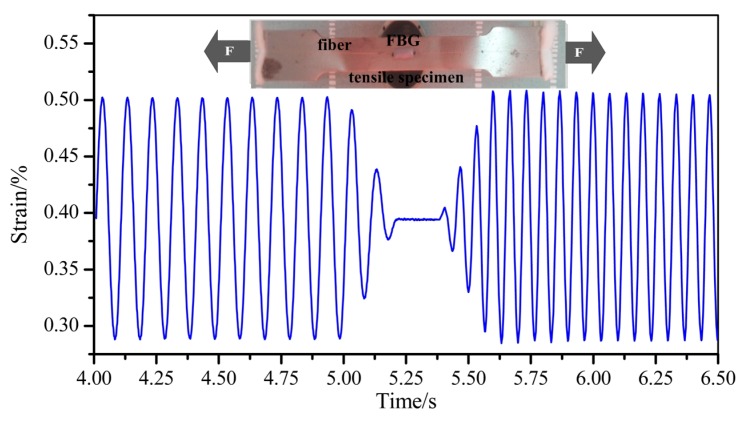
Dynamic strain measurements at 10 Hz and 15 Hz.

Dynamic strain measurements were also carried out. As shown in Figure 9, a metal tensile specimen (300 mm in length and 30 mm in width) was made for a dynamic strain test. FBG3 with a bonding length of 20 mm was fixed to the middle part of the surface using a liquid epoxy resin adhesive. Then, the specimen was loaded with 10 Hz and 15 Hz sinusoidal strain waveforms (2900–5000 µε) sequentially in the MTS universal testing machine. Figure 9 shows the dynamic strain in the time domain. As shown, the dynamic response is in agreement with the two different sinusoidal traces. The loading and unloading processes can also be identified clearly. The dynamic strain can be well-measured by this hybrid system.

## 5. Multiparameter Monitoring of a Slope Based on the Hybrid System

### 5.1. The Hybrid BOTDA/XFG-Based Multiparameter Monitoring System

**Figure 10:**
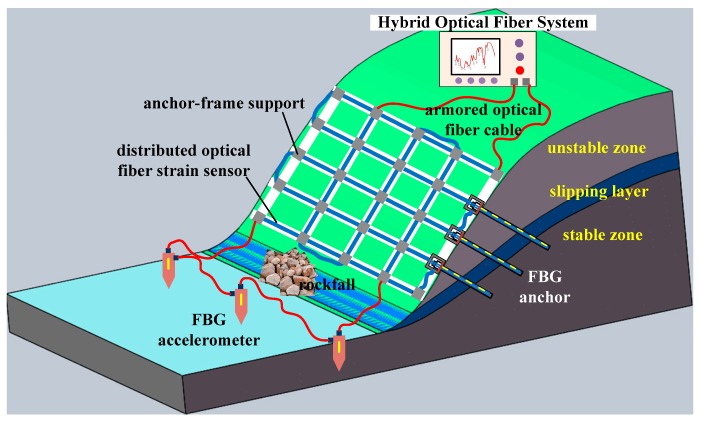
Multiparameter monitoring of a slope using the proposed hybrid system.

In order to determine the steady state of a slope, early detection is essential. Decreases in a slope’s stability are often accompanied by surface and subsurface deformation, increases in the stress on the reinforcement, variation in soil moisture, and rockfall occurrences. Multiparameter monitoring is critical for a landslide warning system to function correctly and in real-time since it can provide more information. Therefore, a scheme for multiparameter monitoring of slopes is developed using the proposed hybrid system, which can monitor static and dynamic information simultaneously. As illustrated in Figure 10, an anchor-frame girder with an embedded optical fiber sensor is designed to monitor surface deformation and anchor force change due to subsurface deformation. At the same time, an optical fiber accelerometer is also introduced to detect vibrations induced by rockfall.

**Figure 11:**
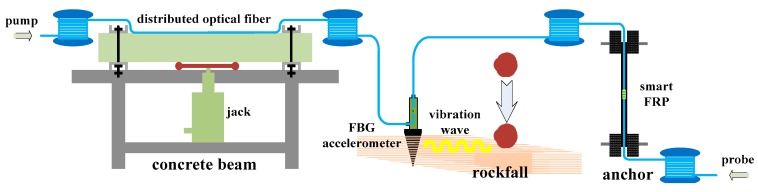
A schematic diagram of the simulated experimental system.

Based on the developed scheme, proof-of-concept experiments have been carried out using the proposed hybrid sensor to verify its feasibility for monitoring multiple parameters of slopes simultaneously. Figure 11 shows the schematic diagram of the simulated experimental system, which contains three parts. The first part simulates the surface deformation of a slope with a distributed fiber sensor bonded to the surface of a concrete beam. Here, we utilized a jack load to replace the push force induced by a landslide. Surface deformation of a concrete beam was studied using a four-point bending test. In the second part, a smart FRP rod [49] with an embedded FBG sensor was loaded using a universal testing machine to simulate variations in the axial force of an anchor reinforcement in a slope. Stones falling from a higher level were used to simulate rockfall on a slope in the third part, where a previously designed FBG accelerometer [50] is adopted to obtain rockfall vibrations. At the same time, a metal nail was used to couple the FBG accelerometer and the surrounding soil or rock.

The above mentioned optical fiber sensors were integrated into the hybrid system and performed measurements simultaneously.

### 5.2. Measurement of Distributed Static Strain on a Frame Beam

**Figure 12:**
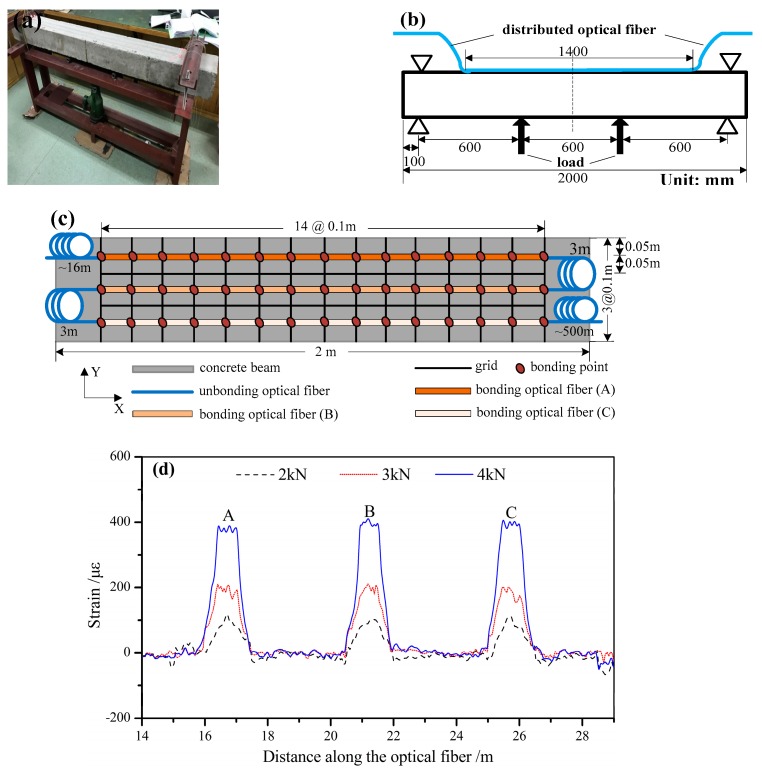
Measurement of the strain distribution in the frame beam: (**a**) the test setup; (**b**) the dimensions of the test setup; (**c**) the layout of the distributed optical fiber sensor; (**d**) the measurement results of the surface strain on the frame beam.

As shown in Figure 12a, an approximate four-point bending test with a distributed optical fiber point-bonded onto the surface of a concrete beam was carried out to simulate variation in frame beam strain induced by surface deformation of a slope. The test was conducted using load control, in which a hydraulic jack and a force sensor are utilized to load a beam and measure loads, respectively. Three load levels were considered: 2 kN, 3 kN, and 4 kN. The dimensions of the test setup are shown in Figure 12b. Figure 12c shows the layout of the distributed optical fiber sensors as well as the subareas of the concrete beam. For ease of reference, the three portions of the point-bonded optical fiber sensor are marked by A, B, and C.

It takes several minutes for the hybrid system to measure the strain distribution along the optical fiber at each step. The Brillouin frequency shift of each portion was directly measured with the hybrid system and converted into a change in strain considering a calibration coefficient of 0.050 MHz/µε. Figure 12d shows the strain distribution of optical fibers A, B, and C under different load levels. The strain distributions measured by A, B, and C were almost identical. The curves are similar to a trapezoid at different load levels, which is basically consistent with the theory of a four-point bending test. However, the measured strain distribution did not agree well with the theoretical results because the boundaries were not strictly fixed. However, the strain value and the spatial recognition notwithstanding, it is sufficient to monitor the surface deformation of a slope.

### 5.3. Measurement of Dynamic Strain on an Anchor

**Figure 13:**
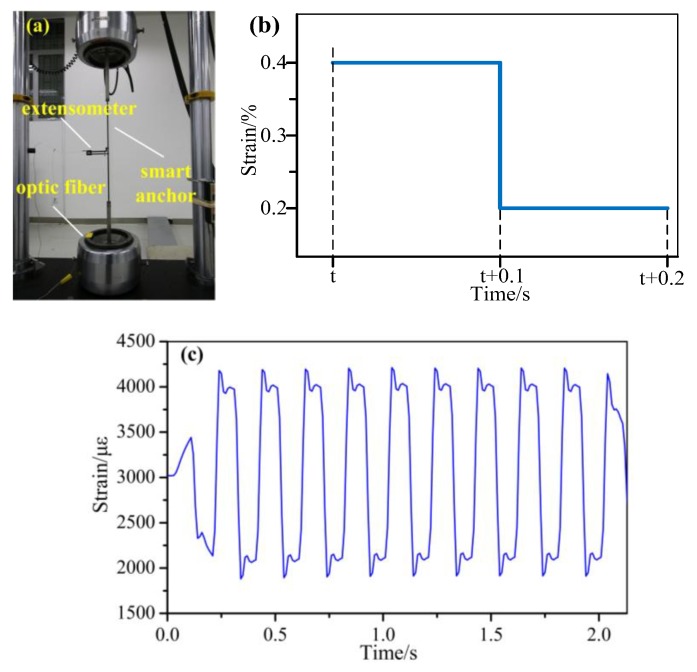
Measurement of the dynamic strain on the anchor: (**a**) the test setup; (**b**) the load with a square waveform applied to the anchor; (**c**) the measurement results of the dynamic strain on the anchor.

An anchor reinforcement plays an important role in keeping a slope steady. Change in the axial force on an anchor reinforcement is also a key index for evaluating a slope’s stability. As shown in Figure 13, a dynamic test on a smart FRP bar was conducted using an MTS universal testing machine to simulate changes in axial force. The smart FRP bar with an embedded FBG sensor was cyclically loaded with a square waveform between 2000 µε and 4000 µε at a frequency of 5 Hz (Figure 13b). The FBG wavelength was recorded by the hybrid system. At the same time, an extensometer with a gauge length of 25 mm was attached to the surface of the smart FRP bar. The time-domain response of the FBG sensor is shown in Figure 13c, which indicates that the hybrid system can record the bar’s strain perfectly under cyclic loading. The wave distortion between Figure 13b and c is due mainly to hysteresis and overloading.

### 5.4. Measurement of Rockfall Vibration

**Figure 14:**
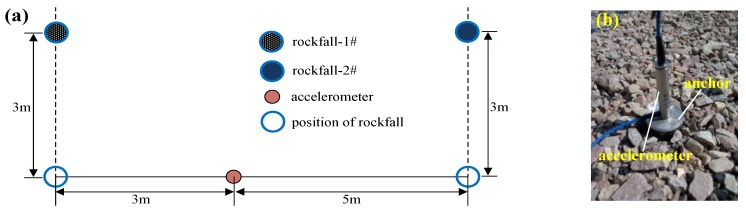

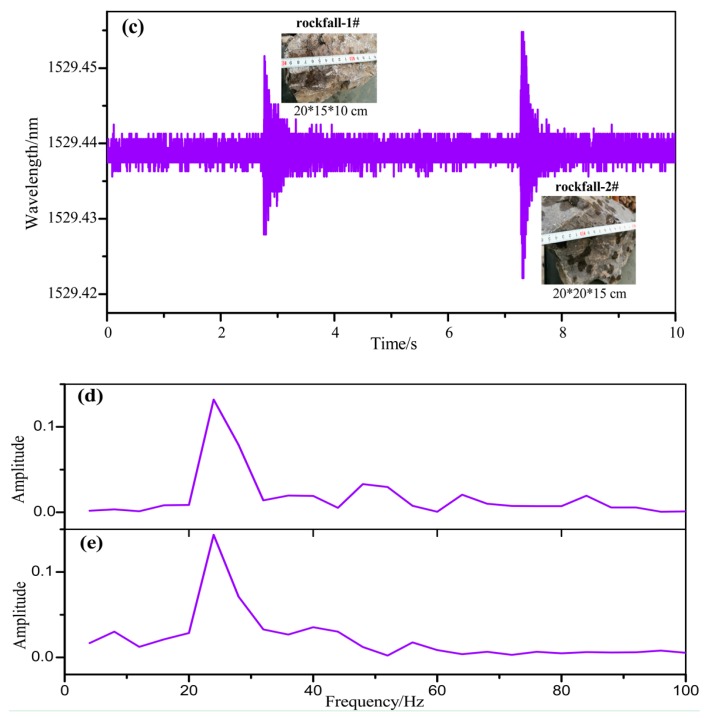
Measurement of rockfall vibration: (**a**) a schematic diagram of the experiment; (**b**) a photo of the fiber Bragg grating (FBG) accelerometer; (**c**) the vibration signal measured in the time domain; (**d**) the frequency response of rockfall-1#; (**e**) the frequency response of rockfall-2#.

Rockfall is one of a landslide’s hazard modes, and is also a precursor to a landslide. Two stones with different sizes freely falling from a higher level were utilized to simulate rockfall. Figure 14 shows the experiment on rockfall monitoring by using an FBG accelerometer and the hybrid system. Rockfall-1# with an approximate size of 20 × 15 × 10 and rockfall-2# with an approximate size of 20 × 20 × 15 were selected for this experiment. They both fell from a height of 3 m above the ground. The position of rockfall-1# was located 3 m from the accelerometer, and the position of rockfall-2# was located 5 m from the accelerometer. Figure 14c illustrates the measured vibration signals that correspond to rockfall-1# and rockfall-2#. It is further verified that the hybrid system has the capability for dynamic measurement. Figure 14d,e show the normalized fast Fourier transform (FFT) of the measured vibration signals. Rockfall-1# and rockfall-2# have almost the same vibration frequency of 25 Hz. The vibration induced by rockfall can be captured clearly by this hybrid system.

## 6. Conclusions

In summary, we have presented a highly integrated hybrid sensing scheme that combines a stimulated Brillouin scattering (SBS)-based distributed sensor with XFG (FBG and LPFG) sensors for fully distributed and multiple discrete dynamic measurements. Based on the experimental results, the following conclusions can be drawn:

(1) A hybrid PPP-BOTDA/XFG sensing system was developed using a shared light source and a single fiber. In order to achieve a better SNR, the wavelength for XFG interrogation is suggested to be located at 1525.60–1547.02 nm and 1550.11–1568.30 nm. By using EDFA, DWDM, and a band-pass filter, the XFG reflected/transmitted signal and the stimulated Brillouin scattering signal can be measured simultaneously without interference.

(2) Dynamic strain and distributed temperature tests were conducted with an MTS universal testing machine and a TCC. The experimental results show that both Brillouin-based distributed temperature measurement and FBG-based quasi-distributed dynamic strain measurement can be achieved simultaneously.

(3) A scheme for multiparameter monitoring of slopes was developed using this hybrid system. Its feasibility for the simultaneous monitoring of surface deformation, anchor reinforcement, and rockfall vibration in a slope was verified in a simulation experiment.

(4) To improve this hybrid system’s performance, effort should be made in three respects: the XFG sensor’s sensing range, the multiplexing type, and applications.

① Raman amplification may be a good choice for the improvement of the sensing range of XFG sensors.

② Considering that LPFG sensors are more sensitive to humidity, a soil moisture sensor based on LPFG sensors should be developed to make the hybrid system more inclusive.

③ Further application of the hybrid system is needed to quantify the uncertainty in its measurements in practice.
